# Protein quality and glycemic indexes of mango drinks fortified with a soybean/maize protein isolate with three levels of urease activity fed to weanling rats

**DOI:** 10.29219/fnr.v66.8576

**Published:** 2022-11-11

**Authors:** Katia Caballero-de la Peña, Laura Acevedo-Pacheco, Aidee I. Sánchez-Reséndiz, Cristina Chuck-Hernández, Sergio O. Serna-Saldívar

**Affiliations:** 1 Tecnologico de Monterrey, School of Engineering and Sciences, Monterrey, México; 2 Tecnologico de Monterrey, The Institute for Obesity Research, Monterrey, México

**Keywords:** soy protein isolate, glycemic index, beverages, mango, protein digestibility, urease activity, artificial sweeteners

## Abstract

**Introduction:**

Public health professionals established a direct link between obesity and the rise in high caloric beverage intake. Current recommendations promote the elimination of sweet fruit drinks from the population’s diet. One way of evading this is by modifying the drink’s nutritional characteristics regarding nutrient uptake and utilization.

**Objectives:**

evaluate the protein quality of a soy/maize protein (SMP) and its physiological effects on nutrient intake and to assess glycemic indexes (GIs) of mango based drinks prepared with sucrose or stevia.

**Materials and methods:**

Mango drinks were supplemented with different sources of protein (three SMP thermally treated to contain different urease activities (UA) or whey protein concentrate (WPC)) that were sweetened with sucrose or stevia/sucralose. The protein digestibility, net protein absorption (NPA), biological value (BV), net protein utilization (NPU) value and protein efficiency ratio (PER) were assessed with weanling rats. Moreover, the GIs of the mango drinks were measured in the same animal model.

**Results:**

PER and NPA evaluated in a rat model showed that increased levels of UA decreased Biological (BV) and Net Protein Utilization (NPU) values. The GIs of the mango drinks significantly diminished with the addition of 3.5% of SMP, but unexpectedly the substitution of sucrose by stevia/sucralose did not significantly change the glycemic response.

**Conclusion:**

the SMP isolate can be used to improve the nutritional profile and lower GIs of mango drinks.

## Popular scientific summary

- In recent years, obesity has increased dramatically, associated with increased beverage intake with high caloric load and high glycemic index.
- Mango drinks fortified with different protein sources have significantly reduced glycemic indicators.
- This work assessed different parameters of mango-based drinks supplemented with whey protein isolate or novel soy/maize protein isolates sweetened with sucrose or stevia, aiming to explore the development of a healthier beverage for the population.

The incidence of obesity has risen dramatically in the last couple of decades, being diabetes one of the main complications associated with this physiological disorder. Public health professionals established a direct link between obesity and the rise in high caloric beverage intake, both in children and adults (1). Therefore, current recommendations promote the elimination of sweet fruit drinks from the population diet (2). One way of evading this is by modifying the drink’s nutritional characteristics regarding nutrient uptake and utilization.

We propose modifying the glycemic index (GI) by supplementing mango drinks with a combination of a legume/cereal protein isolate. In theory, this combination yields a high-quality protein with biological characteristics similar to casein and whey protein concentrate (WPC) at lower costs. As far as these authors know, only one beverage was reported comprised of a soy/maize combination. This beverage had dairy traits instead of those commonly associated with mango drinks. In addition, it had an *in vitro* protein digestibility (IVPD) higher (88%) than its soy counterpart (82%) and Protein Efficiency Ratio (PER) values that correlated favorably with casein (3). Thus, the legume/cereal protein isolate used in this study will have the sought-after nutritional characteristics of animal proteins, with the benefit of being affordable to people from different socioeconomic levels.

Despite the high protein quality and low cost of a soy/maize blend, its high content of anti-nutritional factors due to an improper thermal treatment reduces its *in vivo* bioavailability (compared with WPC and casein) by reducing the catalytic action of key digestive enzymes such as trypsin (4–6).

The objective of this study was to evaluate the protein quality of the soy/maize protein (SMP) and its physiological effects on nutrient intake and to assess GIs of drinks prepared with sucrose or stevia. This was achieved by formulating a mango fruit drink sweetened with the two different sweeteners and supplemented with 3.5% SMP with varying degrees of UA (1.89, 0.66, and 0.17). It was hypothesized that the gradual reduction of UA would improve its protein quality to values similar to those of animal proteins. To achieve these objectives, both an *in vitro* and *in vivo* weanling rat models were used to assess differences in protein digestibility, nitrogen retention, animal growth, and GI among treatments.

## Materials and methods

### Protein sources

Casein (MP Biomedicals, USA), WPC (Hegart S.A. de C.V., México), and SMPs (Industrias Nutrigrains, México) contained 86, 80, and 84% of protein, respectively. To produce the low UA SMP (0.17), an additional thermal treatment during the process was applied (Industrias Nutrigrains, Mexico). This was due to preliminary findings that showed low protein bioavailability due to the high content of antinutritional compounds.

### Mango drinks formulation

Four mango fruit drinks were developed using a commercial formulation from a local food company. Two SMP mango drinks were formulated: one sweetened with sucrose (SMPS) and another with a combination of stevia/sucralose (SMPSS). As references, two additional beverages were developed: one supplemented with WPC 80 and another one without protein. All mango drinks contained the following ingredients: mango pulp (Del Valle, México), citric acid (Desarrollo de comerciales S.A. de C.V., México), sucralose (SW Food Technology S.A. de C.V., México), standard sucrose (Hill Country Fair, USA), stevia (Metco®, México), antifoam Fermcap (Kerry Ingredients and Flavours, Ireland), orange-red dye (Sabores, colores y perfumes S.A. de C.V., México), ethylenediaminetetraacetic acid (EDTA) (Innovadora de alimentos y químicos S. de R.L., México), and sodium tripolyphosphate (STPP) (Proveedores de ingeniería alimentaria S.A. de C.V., México). Formulations for each beverage are depicted in Table 1. The beverage industry routinely uses these additives in its formulations. Citric acid provides flavor balance between acids and sweeteners (7); ascorbic acid and STPP extend shelf life by promoting unfavorable environments for microbial growth (8), whereas EDTA, STPP, and ascorbic acid enhance product quality and stability (9, 10). All mango drinks were prepared in a high-velocity mixer (Ultraturrax T18 Basic S1 IKA, Germany) at 14,000 rpm for 5 min. Pasteurization was performed at 80ºC for 30 sec.

**Table 1:** Formulation and ingredients of mango fruit drinks

	Mango fruit drink (%)	Mango fruit drink with WPC^c^ (%)	Mango fruit drink with SMP^d^ and sucrose (%)	Mango fruit drink with SMP and stevia/sucralose (%)
Mango pulp	8.42	8.42	8.42	6.00
Water	84.1	83.1	83.1	88.4
Citric acid	0.50	0.50	0.50	0.80
Ascorbic acid	0.01	0.01	0.01	0.01
Protein	0.00	3.50	3.50	3.50
Sucralose	0.02	0.02	0.02	0.04
Sucrose	6.90	3.60	3.61	0.00
Stevia	0.00	0.00	0.00	0.50
Antifoam	0.30	0.30	0.30	0.16
Dye	0.004	0.004	0.004	0.004
EDTA^a^	0.02	0.02	0.02	0.02
STPP^b^	0.13	0.13	0.13	0.13
Total	100	100	100	100

a Ethylenediaminetetraacetic acid;

b sodium tripolyphosphate;

c whey protein concentrate;

d soy/maize protein.

### *In vitro* protein digestibility

IVPD was measured in triplicate using the pH-based Multi-Enzyme Technique suggested by Hsu et al. (11) with porcine pancreatic trypsin (Sigma T4799), bovine pancreatic chymotrypsin (Sigma C4129), and *S. griseous* protease type XIV (Sigma P5147), the latter as a substitute for porcine intestinal peptidase according to Hervera et al. (12). IVPD was calculated using the following equation:


IVPD = 210.46 −180.10 (final pH) (Equation 1)

### Diets for *in vivo* studies

Five diets were prepared varying the source of protein (casein, WPC, SMP (1.89 UA), SMP (0.66 UA), and SMP (0.17 UA)). All diets were formulated with the mango drinks’ ingredients described in Table 1, using them as a unique source of protein (10%). Besides the solids from mango drinks, the following ingredients were included in all the formulations: 8% corn oil (Cristal^®^, México), 1% vitamins and 3.5% minerals (MP Biomedicals, USA), 1% cellulose (Desarrollo de Especialidades Quimicas, México), and native corn starch (Maizena®, México) in the following percentages: 68.2% for casein diet, 36.5% for WPC, and 47.5% for all SMP treatments. Diet formulations were thoroughly blended in a Hobart 5 Lt mixer equipped with a paddle attachment for 10 min (SM-30; Hobart, USA).

### *In vivo* protein quality assay

The PER procedure (13) was used to assess the protein quality of the different mango drinks in five experimental groups with a minimum of six rats each. The study groups were set according to the diets previously described. Weanling male Wistar rats weighing 40–45g were individually housed in metabolic cages (<300g, Nalgene) for 28 days. The cages were equipped with a collector that separates the fecal pellets from the urine. The urine collection containers were supplemented with 0.5 mL of 1:1 HCl:water to prevent microbial degradation. The feces and urine were collected daily during the last 15 days of the study and immediately placed under frozen storage (–20°C). Feces were dehydrated at 60°C overnight before grinding and analysis, whereas the urine was brought to a fixed volume with distilled water before analysis. Food and water were supplied *ad libitum*. Nitrogen determination in diets, feces, and urine was assayed using the micro Kjeldahl method AOAC 978.02 (13).

### Protein quality values

Using the data collected from the *in vivo* study, the following values were calculated according to the equations from (14):


$$PER:\frac{weight\;gain\;(g)}{p\mathrm{rotein}\;\mathrm{intake}\;(g)}$$ (Equation 2)

$$\mathrm{BV}:\;\frac{N_{2}\;\mathrm{food}\;-\;N_{2}\;\mathrm{feces}-N_{2}\;\mathrm{urine}}{N_{2}\;\mathrm{food}-N_{2}\;\mathrm{feces}}$$ (Equation 3)

$$\mathrm{APD}:\;\frac{N_{2}\;\mathrm{food}\;-\;N_{2}\;\mathrm{feces}}{N_{2}\;\mathrm{food}}$$ (Equation 4)

$$\mathrm{NPU}:\frac{\mathrm{APD}*\mathrm{BV}}{100}$$ (Equation 5)

$$\mathrm{NPR}:N_{2}\;\mathrm{food}–N_{2}\;\mathrm{feces}–N_{2}\;\mathrm{urine}$$ (Equation 6)

$$\mathrm{NPA}:N_{2}\;\mathrm{food}–N_{2}\;\mathrm{feces}$$ (Equation 7)

Protein Digestibility Corrected Amino Acid Score


$$\mathrm{PDCAAS}=\frac{\mathrm{APD}*\mathrm{score}\;\mathrm{of}\;\mathrm{limiting}\;\mathrm{amino}\;\mathrm{acid}}{100}$$ (Equation 8)

For the last value, essential amino acid contents of casein, WPC, and SMP were determined (Table 2) using the AOAC 982.3 method (13).

**Table 2:** Essential amino acid composition and percentage of contribution to preschool children’s diet according to the FAO/WHO recommendations of SMP, casein, and WPC

Amino acid	FAO/WHO^a^ g/100g P^e^	SMP^b^ g/100g P^e^	%R^c^	Casein g/100g P^e^	%R	WPC^d^ g/100g P^e^	%R
Threonine	3.4	2.49	73.24	3.36	98.82	4.40	129.41
Valine	3.5	3.31	94.57	5.43	155.14	3.82	109.14
Methionine/cysteine	2.5	1.67	66.80	3.15	126.00	2.76	110.40
Isoleucine	2.8	3.27	116.79	2.61	93.21	3.93	140.36
Leucine	6.6	5.52	83.64	7.78	117.88	6.92	104.85
Phenylalanine/tyrosine	6.3	6.49	103.02	9.07	143.97	4.24	67.30
Lysine	5.8	4.27	73.62	6.44	111.03	5.77	99.48
Histidine	1.9	1.81	95.26	2.46	129.47	1.20	63.16
Tryptophan	1.1	1.01	91.82	1.17	106.36	1.62	147.27

a Food and Agriculture Organization/World Health Organization;

b soy/maize protein;

c requirement;

d whey protein concentrate;

e protein.

### In vivo GI determination

GI calculations were done following the modified method of the human GI determination ISO 26642 (15). Five study groups were used with five rats each: 1) mango drink without added protein, 2) mango drink with WPC and sucrose, 3) mango drink with SMPS, 4) mango drink with SMPSS, and 5) stevia ingredient. 1.5 g of glucose/kg body weight after 12 hr of fasting was delivered using intragastric probes (16). Blood glucose measurements were performed with a glucometer (OneTouch Ultra Mini, Johnson Medical^®^) by triplicate at 0, 15, 30, 45, 60, 90, and 120 min after mango drink ingestion. GI values were obtained with the following equation: $\mathrm{GI}=\frac{\mathrm{AUC}\;\mathrm{of}\;\mathrm{the}\;\mathrm{fruit}\;\mathrm{drink}}{\mathrm{AUC}\;\mathrm{of}\;\mathrm{glucose}}\times 100$ (Equation 9) (AUC: area under the curve). Values were compared to the standard GI classification: low (<55), medium (55–69), and high (>70) (17).

### Statistical analyses

Data were statistically analyzed using ANOVA (MiniTab statistical software v. 17 Minitab Ltd., Coventry, UK) procedures. Significant means differences were determined using Tukey’s test (*P* < 0.05).

## Results and discussion

### *In vitro* protein digestibility

The IVPD of the individual proteins showed that casein had the best digestibility, followed by the WPC and thermally treated SMP (Table 3). Results obtained from this study were similar to the IVPD reported by Hsu et al. (11) for casein (100%) and soy (88.1%).

**Table 3:** *In vitro* protein digestibility of individual proteins

Protein	*In vitro* digestibility (%)
Casein^a^	97.61 ± 2.81a
WPC^b^	90.16 ± 1.93 b
SMP^c^ (non-thermal treated)	85.51 ± 1.61 c
SMP (thermal treated)	89.13 ± 0.10 b

The values shown in this table are the average ± standard deviation.

Values within columns with the same letter are not significantly different at *P* < 0.05.

a Casein;

b whey protein concentrate;

c soy/maize protein.

Results for WPC were higher than those reported by Hsu et al. (11) (76.4 %). This discrepancy may be due to differences in the source of WPC. It has been reported that differences in physical and chemical properties, as well as the country of origin or drying and pasteurization methods, can affect the nutritional quality of a WPC (18–20).

**Fig. 1:**
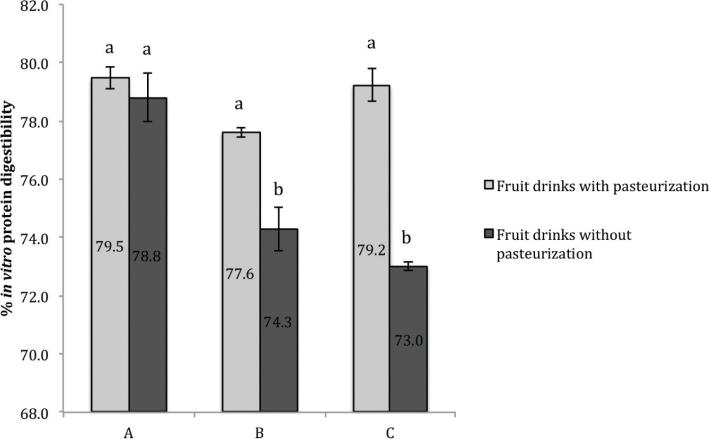
*In vitro* protein digestibility of mango fruit drinks. Bars within a column with the same letter are not significantly different at *P* < 0.05. (A) Mango fruit drink with whey protein concentrate; (B) mango fruit drink with soy/maize protein with sucrose; (C) mango fruit drink with soy/maize protein with stevia and sucralose.

Figure 1 shows a statistical similarity between the SMP and WPC supplemented mango drinks, but after comparing these results with those obtained for individual SMPs (Table 3), a decrease in IVPD was observed. Two possible causes were considered: 1) pasteurizing the mango drinks negatively affected the IVPD; 2) a protein-ingredient interaction reduced the protein’s digestibility. After performing the IVPD determination on unpasteurized mango drinks, the first hypothesis was discarded because an improvement in SMP digestibility was observed when the mango drinks were pasteurized (Fig. 1). Consequently, IVPD determinations of SMP with each mango drink’s ingredients were done. These results indicate that all the ingredients lower the IVPD to some extent, being acids, mango pulp, and STPP the predominant IVPD lowering ingredients (6.4, 4.16, and 3.68%, respectively).

### *In vivo* protein quality determination

**Fig. 2:**
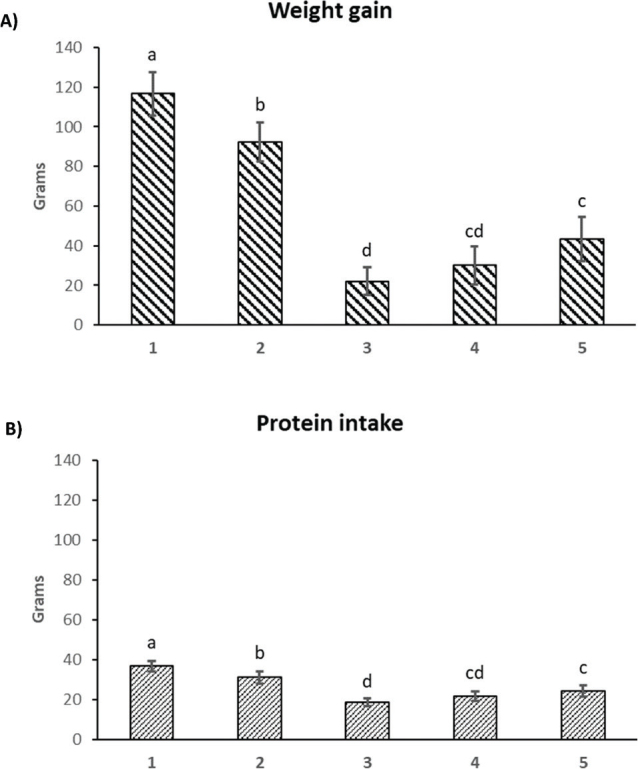
Weight gain and protein intake of study groups. Bars within a column with the same letter are not significantly different at *P* < 0.05. (1) Casein diet; (2) whey protein concentrate diet; (3) soy/maize protein (1.89)* diet; (4) soy/maize protein (0.66)* diet; (5) soy/maize protein (0.17)* diet; *urease activity.

Figure 2 compares weight gain and protein intake for the five studied groups. It is clear that the quality of the SMP increased as the content of UA decreased. At relatively the same amount of protein intake, animals fed the SMP (0.17 UA) gained more weight than counterparts fed the SMP containing 1.89 or 0.66 UA. In addition, El-Niely (21) and Olguin et al. (22) researched the inactivation of anti-nutritional compounds in soybean protein and observed an increase in food intake and weight gain as the anti-nutritional compounds in the diet were inactivated. The reduction of the SMP’s UA increased 96% weight gain.

Nitrogen metabolic values depicted in Table 4 showed that the low UA SMP diet had superior PER, APD, and Net Protein Utilization (NPU) values compared to the diet containing high UA. These are indicators of weight gain related to protein intake, protein digestibility, and how much of the digested protein was absorbed and retained by the body. As UA was reduced, an increase in 38% of the PER value was achieved. Interestingly, both NPU and Biological Value (BV) decreased as UA in the SMP increased. These values are known to be highly affected by the bioavailability of the protein’s limiting amino acid. Additionally, we observed that even though the vegetable proteins with low UA doubled rat weight gains and significantly increased PER values, they were still comparatively lower than dairy proteins casein and WPC. Wu et al. (23) reported a decrease in *in vivo* protein digestibility due to lower bioavailability of specific amino acids related to increased times and temperatures of thermal treatments in red kidney beans. The percentage of essential amino acid contributions based on the FAO/WHO recommendation for preschool children was calculated to corroborate this theory. These results conclude that the low BV and NPU values were due to inadequate content of lysine, threonine, and methionine (73, 73, and 67% of the requirement, respectively) (Table 2). Another cause for the reduction of BV and NPU values and the moderate reduction in weight gain may be the residual content of antinutritional factors in the protein, specifically trypsin inhibitor, which blocks the active site of the pancreatic enzyme trypsin reducing protein digestibility (24).

**Table 4:** Protein quality values obtained from the *in vivo* assay

Treatments	PER^c^	BV^d^ (%)	APD^e^ (%)	NPU^f^ (%)	NPR^g^	NPA^h^	PDCAAS^i^ (%)
Casein	2.50 ± 1.19a	69.59 ± 8.30a	91.34 ± 2.34a	63.61 ± 8.05a	2.02 ± 0.40a	2.90 ± 0.49a	0.95
WPC^a^	2.32 ± 0.12a	72.00 ± 4.79a	86.70 ± 1.79b	62.43 ± 4.42ab	1.72 ± 0.25a	2.39 ± 0.25b	0.68
SMP^b^ (1.89)*	0.92 ± 0.17c	64.87 ± 12.00ab	82.69 ± 1.45c	53.59 ± 9.61bc	0.82 ± 0.16b	1.27 ± 0.17d	0.66
SMP (0.66)*	1.16 ± 1.56c	57.57 ± 4.41b	82.69 ± 2.15c	47.56 ± 3.21c	0.92 ± 0.10b	1.61 ± 0.22cd	0.66
SMP (0.17)*	1.48 ± 0.31b	54.79 ± 7.87b	83.87 ± 1.33bc	45.93 ± 6.38c	0.99 ± 0.29b	1.78 ± 0.31c	0.67

The values are the average ± standard deviation. Data within a column with the same letter are not significantly different at *P* < 0.05.

* Urease activity;

a whey protein concentrate;

b soy/maize protein with sucrose;

c protein efficiency ratio;

d biological value;

e apparent protein digestibility;

f net protein utilization;

g net protein retention;

h net protein absorption;

i protein digestibility corrected amino acid score.

PER values for the SMP obtained herein depicted similarity to those reported by Serrem et al. (25) for a protein that consisted of a combination of sorghum and soybean. However, Egounlety et al. (26) managed to obtain a higher quality protein from a combination of soy/maize with PER values similar to those obtained for casein. These authors obtained this improvement in protein quality due to the addition of melon seed flour to the final formulation. Melon seed flour is high in methionine, the limiting amino acid of soybean.

*In vitro* PDCAAS and *in vivo* PER values are commonly used to measure food protein quality (27). Casein (1.00) results are similar to those reported by Sarwar (27). The SMP PDCAAS obtained in this study is higher than a traditional and a thermally treated soy protein isolate (0.62 and 0.44, respectively) but lower than milk (0.74) (27). There are no PDCAAS values published for a protein consisting of a soy/maize mixture, but results from this study clearly state that even though they are higher than a soy protein isolate’s, this combination is not enough to completely cover or meet the essential amino acid requirements for preschool children. Regarding WPC, results were considerably lower than what is found in the literature; this is because the amino acid histidine limited the value of this protein source.

Results herein showed that the SMP’s quality strictly depends on both urease activity and the concentration of sulfur-containing amino acids (methionine/cysteine). It was observed that reducing anti-nutritional compounds improved protein quality to a certain extent. However, low PER values observed for the SMP with low urease activity were due to its limited content of methionine/cysteine (66.8% of the recommendation for infants). Therefore, the recommendation is to use a mixture of SMP and WPC to produce a high-quality protein. WPC will provide the sulfur amino acids limiting in the SMP, and SMP will provide the aromatic amino acids (phenylalanine/tyrosine) that WPC is deficient on.

### *In vivo* GI determination

Even though the effect of protein in the GI is widely known, most of these studies have been performed with animal proteins. Because of the unique characteristics of this legume/cereal protein, the GI of the mango drinks was measured. It was decided that the mango drink would be supplemented with 3.5% of SMP to emulate a dairy beverage’s nutritional characteristics. The GIs obtained from both SMPS and SMPSS were close to those reported by Brindal et al. (28) for whole milk. Concluding that even though SMP does not have the same protein quality as an animal protein, it has the same physiological effects on glucose uptake.

There were no significant differences between the two samples regarding the SMP mango drinks with different sweeteners. Therefore, it was hypothesized that the substitution of sucrose for stevia/sucralose would aid in the reduction of the mango drink’s GI. To ascertain that the artificial sweetener stevia did not produce a GI response, the GI of stevia was measured. Surprisingly, the results showed that stevia exerted a glycemic response, even though it provided 89% fewer calories than sucrose (data not shown).

Research is still being done on the health effects of consuming stevia and its derivatives. Most authors coincide with an antihyperglycemic effect due to the stimulation of insulin secretion in both *in vitro* and human and animal studies (29, 30). Studies in humans have reported a decrease in blood glucose levels after an acute administration of steviosides. These results were dose dependent, with doses higher than what an average person would consume on any given day (31). Currently, Suanarunsawat et al. (32) are the only authors to witness a hyperglycemic effect of steviosides in normal rats. These authors concluded that this effect might be due to a suppression of insulin-induced glucose uptake by diaphragm muscles.

On the other hand, the administration of steviosides did not affect blood glucose levels in streptozotocin-induced diabetic rats. Interestingly, they observed that the supplementation of stevia extracts to diabetic rats had antihyperglycemic effects. These authors theorize that the steviosides do not provide antihyperglycemic protection, and that another glycoside present imparts this characteristic in the stevia extract. Results from this study differ from what Jeppesen et al. (29, 31) and Anton et al. (30) reported but are consistent with Suanarunsawat et al.’s (32) findings, in which the hyperglycemic effect of steviosides, in part, may be due to a glycemic response when ingested and its consequent metabolic alterations, especially considering that, because of its caloric content, stevia extracts should not be producing a glycemic response. Because of these controversial results, further research is needed to determine the glycemic response pathway of the consumption of stevia and other non-caloric sweeteners.

**Fig. 3:**
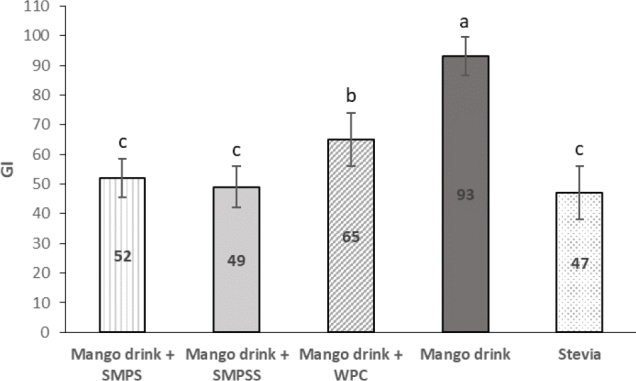
Glycemic index values for mango drinks supplemented with protein and artificial sweeteners. Bars within a column with the same letter are not significantly different at *P* < 0.05. SMPS: soy/maize protein with sucrose; SMPSS: soy/maize protein with stevia and sucralose; WPC: whey protein concentrate.

## Conclusions

Even though, in theory, the SMP is of high BV, in practice, we have shown that there are additional factors to consider other than pure protein and amino acid contents when a legume/cereal protein is being used as a food component. Suppose the legume protein is not given a proper thermal treatment to inactivate its anti-nutritional compounds. In that case, the body will not anabolize the amino acids from the dietary protein to the same extent, and additional health issues will arise due to the presence of antinutritional factors such as trypsin inhibitor, urease, and hemagglutinins. If the protein is thermally treated in the presence of reducing sugars, it will lower its amino acid bioavailability and protein quality. A mango drink with 3.5% of this protein significantly reduced GI values, thus classifying it as safe for consumption by both obese and diabetic populations. Surprisingly, substituting sucrose with stevia did not significantly affect the glycemic response in laboratory animals. Therefore, further research is needed to comprehend the metabolic response of non-caloric sweeteners commonly used to prepare dietetic beverages.

With the results obtained herein, the complementation of SMP with either an animal protein or another food source with a high content of sulfur-containing amino acids is advocated. Thus, a low-cost, high-quality mango drink can be produced for the obese and diabetic population. Furthermore, adding this protein to commercial mango drink formulations will be a valuable tool for the battle against obesity.

## Conflict of interest and funding

The authors have not received any funding or benefits from industry or elsewhere to conduct this study.
